# Approaches to quantifying acceptability of pharmaceutical interventions for neglected tropical diseases: A scoping review

**DOI:** 10.1371/journal.pntd.0014272

**Published:** 2026-05-18

**Authors:** Claudia Duguay, Katarina Ost, Alison Krentel

**Affiliations:** 1 Bruyère Health Research Institute, Ottawa, Ontario, Canada; 2 School of Epidemiology and Public Health, University of Ottawa, Ottawa, Canada; Anesvad Foundation, SPAIN

## Abstract

Measuring acceptability is important in the context of pharmaceutical and public health interventions, since it can have an impact on uptake – a factor that extends beyond the safety and efficacy of the intervention. This scoping review aimed to explore how the acceptability of pharmaceutical interventions for neglected tropical diseases has been quantitatively measured in the literature to inform advancement towards a standardized methodology. A systematic search across five databases identified 1340 articles, of which 40 met the inclusion criteria. Twenty articles (50%) were published in the last five years, between 2020 and 2025. Fourteen articles measured acceptability using multiple questions, with nine establishing a threshold for acceptability. Twenty-two articles assessed acceptability using a single question, while four articles reported measuring acceptability but did not provide details about their methodology. Notably, mass drug administration programs targeting preventive chemotherapy for neglected tropical diseases comprised most studies (18/40), yet these studies utilized 9 distinct approaches to measure acceptability, despite their similar implementation strategies. Nearly a third of the articles (13/40) stated that they measured acceptability, however their methodology revealed that they were measuring coverage (received treatment) and compliance (swallowed treatment). Given that acceptability is one of the seven considerations informing World Health Organization guideline recommendations, a standardized method to quantify this multifaceted attribute is essential. As more studies on acceptability are being published in recent years, the lack of standardization becomes increasingly concerning. This review underscores the urgent need for such methodologies to generate reliable, comparable data for research and policymaking.

## Author summary

Acceptability is a critical factor influencing the uptake and success in neglected tropical disease (NTD) interventions, yet it remains inconsistently defined and measured. This scoping review examined 40 studies that quantitatively assessed the acceptability of interventions for NTDs. The findings revealed significant variability in methodologies, with most studies relying on single-question assessments or lacking transparency in measurement approaches. Alarmingly, over half of the twenty-one recognized NTDs lacked any studies addressing acceptability for their current treatment regimens, despite their availability and widespread use. These gaps highlight the need for a standardized approach to measuring acceptability to support the requirements for guideline development set out by the World Health Organization (WHO), enhance comparability across studies, improve intervention uptake and adherence, and guide future research. Addressing these inconsistencies is essential for achieving the targets outlined in the WHO 2030 NTD roadmap and ensuring the successful implementation of pharmaceutical interventions in vulnerable populations.

## Introduction

Acceptability is one of the seven key factors influencing the direction and strength of a recommendation in World Health Organization (WHO) guideline development, alongside quality of evidence, values and preferences, balance of benefits and harms, resource implications, priority of the problem, equity and human rights, and feasibility [1]. Despite its importance, acceptability remains a challenging concept to define and measure effectively. Recent efforts have been made to conceptualize and define treatment acceptability [2–4], with Sekhon et al. (2017) proposing the following definition, “Acceptability is a multi-faceted construct that reflects the extent to which people delivering or receiving a healthcare intervention consider it to be appropriate, based on anticipated or experienced cognitive and emotional responses to the intervention” [4]. While there is growing interest in assessing acceptability, a standardized quantitative method has yet to be widely used.

It is estimated that neglected tropical diseases (NTDs) affect approximately 1 billion people [5]. NTDs are a group of 21 diverse diseases and conditions caused by a variety of pathogens, predominantly affecting people living in vulnerable circumstances. Measuring acceptability is especially important in the context of NTD interventions, since it can have an impact on uptake – a factor that extends beyond the safety and efficacy of the intervention [6–8]. The WHO 2030 NTD roadmap (*Ending the neglect to attain the Sustainable Development Goals: a roadmap for neglected tropical diseases 2021–2030*), which outlines global targets and milestones to prevent, control, eliminate or eradicate NTDs, mentions the term “acceptability” only once in its 196 pages. It states that “achieving the targets outlined in this road map will require consistent emphasis on the availability, accessibility, acceptability and affordability of NTD medicines and other health products and commodities of assured quality” [9]. However, the roadmap does not provide guidance on how to measure or assess acceptability, leaving a critical gap in understanding how to effectively integrate this factor into achieving the outlined targets.

Quantitatively measuring a subjective attribute presents a fundamental challenge, as individual perceptions, experiences, and interpretations can vary widely. This variability makes it difficult to assign precise numerical values that fully capture the nuance and complexity of the concept. These challenges are evident in the measures used to assess the acceptability of NTD interventions. In the published literature, the acceptability of medicines used in mass drug administration (MDA) for the elimination of lymphatic filariasis (LF) has been evaluated using at least two different approaches: 1) a composite acceptability score of nine acceptability indicators [10–12] and 2) the proportion of individuals that ingested the medicines [13]. This scoping review aims to review how acceptability of pharmaceutical interventions for the prevention and treatment of NTDs has been measured and analyzed in the literature to advance towards a standardized approach for measuring acceptability.

## Methods

### Protocol and registration

To conduct and report this scoping review, we followed the PRISMA-ScR (Preferred Reporting Items for Systematic Reviews and Meta-Analysis extension for Scoping Reviews) (S1 File PRISMA-ScR Checklist) [14]. A detailed published protocol is available on Protocol.io [15].

### Search strategy

A systematic search of five academic databases (Medline (Ovid), EMBASE, SCOPUS, Global health and CINAHL), identified in consultation with a librarian at the University of Ottawa, was conducted on November 19, 2025. A detailed search strategy for each database was also designed and piloted in consultation with the librarian. We searched for each NTD (Table 1) using a combination of Medical Subject Heading (MeSH) terms and free-text terms that included both scientific and lay expressions, then refined the search by adding “AND accept*” to include terms related to acceptability. The search strategies for each database are found in the Supplemental Materials (S2 File Search Strategy).

**Table 1:** List of 21 neglected tropical diseases (NTDs) [16].

1	Buruli ulcer
2	Chagas disease
3	Dengue and chikungunya
4	Dracunculiasis
5	Echinococcosis
6	Foodborne trematodiases
7	Human African trypanosomiasis
8	Leishmaniasis
9	Leprosy
10	Lymphatic filariasis
11	Mycetoma, chromoblastomycosis and other deep mycoses
12	Noma
13	Onchocerciasis
14	Rabies
15	Scabies and other ectoparasitoses
16	Schistosomiasis
17	Soil-transmitted helminthiases
18	Snakebite envenoming
19	Taeniasis/cysticercosis
20	Trachoma
21	Yaws

### Screening and study selection

All identified studies were imported into COVIDENCE (Veritas Health Innovation, Melbourne, Australia; available at www.covidence.org), a systematic review management software, to screen (title, abstract, and full text) and manage the results of the search. Two reviewers (CD and KO) independently assessed the titles and abstracts of the included articles based on the inclusion and exclusion criteria (Table 2). Briefly, articles were included if they quantitatively investigated the acceptability of a pharmaceutical intervention for at least one NTD. In the event of discordance between the two reviewers, a third reviewer (AK) resolved any discrepancies. From the included articles, CD and KO identified relevant articles by reviewing the full text. Any discordances were resolved by AK.

**Table 2:** Criteria for inclusion and exclusion.

	Inclusion	Exclusion
**Concept**	• Articles that quantitatively investigates the acceptability of a pharmaceutical intervention for at least one neglected tropical disease	• Articles that investigate non-pharmaceutical interventions (vector control, tests, behavioral interventions). • Articles that investigate the clinical acceptability of a pharmaceutical intervention.
**Type of study**	• Primary research (i.e., clinical trial, cross-sectional survey)	• Secondary research (i.e., systematic reviews, commentary).
**Language**	• Articles in French or English	
**Publication status**	• Articles published or in press	
**Timeline**	• No restriction	

From the included articles, one reviewer (CD) worked independently to extract data from the articles following a pre-specified extraction sheet. The following data were extracted from each article: (1) author; (2) year of publication; (3) study period; (4) study type; (5) year of acceptability study; (6) country of acceptability study; (7) NTD under investigation; (8) intervention being analyzed; (9) working definition for acceptability; (10) instrument used to measure acceptability; (11) method to analyze acceptability; (12) threshold for acceptability; and (13) key findings.

## Results

### Characteristics of reviewed articles

**Fig 1:**
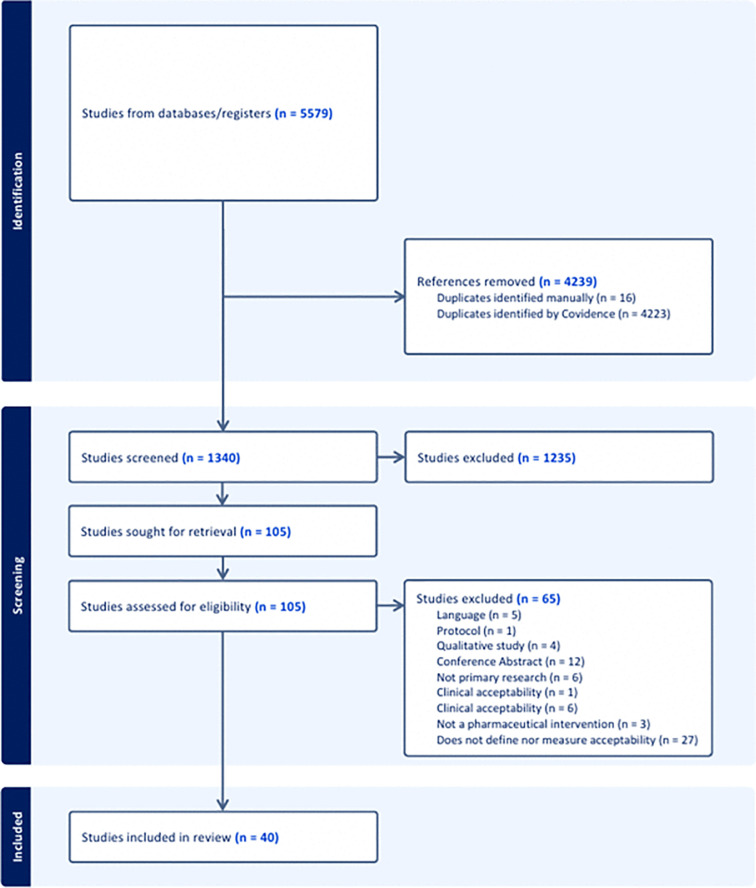
PRISMA flow chart.

The search yielded a total of 1340 articles, after the removal of duplicate records (Fig 1). We excluded 1235 studies upon title and abstract screening, and another 65 articles after full text review.

**Fig 2:**
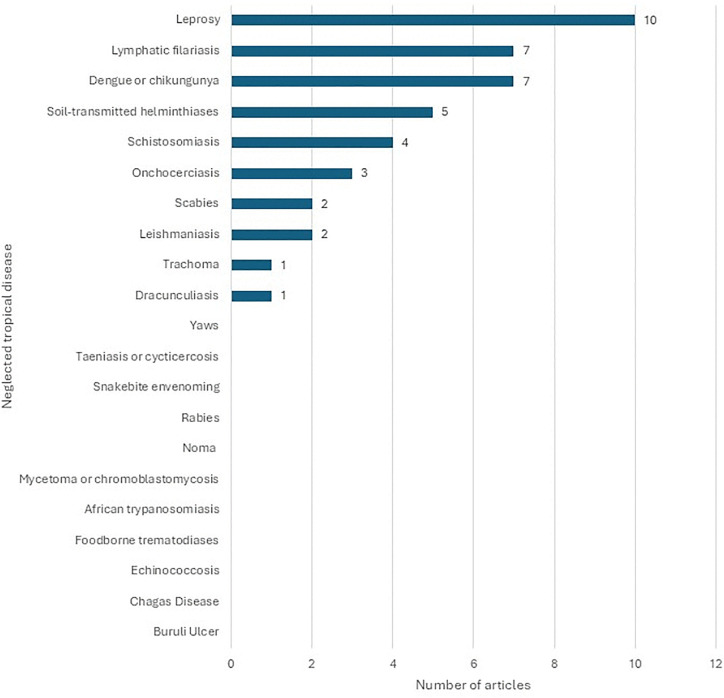
Bar chart depicting the number of articles included in the scoping review for each neglected tropical disease (NTD).

A total of 40 articles were included in the study, with their characteristics summarized in Table 3. Most articles (31, 77.5%) focused on tablets, followed by vaccines (8, 20%) and ointments (1, 2.5%). Half of the articles (20, 50%) were published in the last five years (2020–2025), with the earliest dated in 1978. Only ten of the twenty-one NTDs were represented in the included articles, including: leprosy, lymphatic filariasis, dengue or chikungunya, soil-transmitted helminthiases, schistosomiasis, onchocerciasis, scabies, leishmaniasis, trachoma, and dracunculiasis (Fig 2). Fourteen articles measured acceptability using multiple questions, with nine establishing a threshold for acceptability. Twenty-two articles assessed acceptability using a single question, while four articles reported measuring acceptability but did not provide details about their methodology.

**Table 3:** Characteristics of the articles included in the review.

Author and year	Type of intervention	Number of questions used to measure acceptability	Question(s) used to measure acceptability	Defined threshold of acceptability	Citation
**Leprosy**					
(Manickam et al., 2016)	Tablet	1	Received treatment.	NA	[17]
(Djossou et al., 2025)	Tablet	1	Received treatment.	NA	[18]
(Cartel et al., 1985)	Tablet	1	Received treatment.	NA	[19]
(Mukhopadhyay et al., 2024)	Tablet	1	Received treatment.	NA	[20]
(Kroger et al., 2008)	Tablet	1	Received treatment.	NA	[21]
(Espiridion-Calma et al., 2020)	Tablet	1	Willingness to receive tablet.	NA	[22]
(Cellona et al., 1990)	Tablet	NA	Acceptability of the regimens was determined by the severity of side effects and patient compliance with prescribed drugs. No other information is provided.	NA	[23]
(Faatoese et al., 2016)	Tablet	NA	The authors state that they had a questionnaire on knowledge of leprosy and acceptability of prophylaxis, then report that “an encouraging result was the finding that most respondents would accept chemoprophylaxis against leprosy being offered to family members”. No other information is provided.	NA	[24]
(Zai-Ling et al., 1993)	Vaccine	1	Received vaccine.	NA	[25]
**Lymphatic filariasis**					
(Krentel et al., 2021)	Tablet	9*	Composite score using 9 questions: these drugs work against lymphatic filariasis, these drugs work against itching, these drugs work against intestinal worms, I would take this treatment again, I would recommend this treatment to my relatives, I would be willing to change my family’s routine so that we take the treatment again, I liked this treatment, this treatment is a good way to help our health problems here, and overall, this treatment will help my community.	Yes	[10]
(Niles et al., 2021)	Tablet	9*		Yes	[11]
(Duguay et al., 2024)	Tablet	9*		Yes	[12]
(Pudasainee et al., 2025)	Tablet	9*		Yes	[26]
(Nujum et al., 2012)	Tablet	4	4 questions analyzed independently: sight, cover, number and size.	Yes	[27]
(Amarillo et al., 2008)	Tablet	1	Ingested treatment.	NA	[13]
(Ngunyali et al., 2023)^&^	Tablet	1	Ingested treatment.	NA	[28]
**Dengue and chikungunya**					
(Manaquin et al., 2025)	Vaccine	1	Vaccine acceptability was assessed under three hypothetical scenarios by asking “Would you get vaccinated if the vaccine was…” • recommended by health authorities and free • offered within a clinical trial • officially recommended but not covered, with an estimated cost corresponding to the actual retail price at pharmacies at the time of the study	NA	[29]
(Arham et al., 2022)	Vaccine	7	7 questions analyzed independently: trust in key players, attitudes to technology, religiosity, perceived benefit, perceived risk, attitude and intention to dengue vaccine.	Yes	[30]
(McMahon et al., 2019)	Vaccine	9	9 questions analyzed independently: vaccines are beneficial in improving health, vaccines are safe, a dengue vaccine would be useful in your community/country, you would get a dengue vaccine, you would vaccinate your child(ren) with the dengue vaccine, you would get vaccinated if protection from illness was for less than two years, dengue vaccines should be a standard vaccination, there will be political opposition to the dengue vaccine’s implementation, you would be willing to pay for the dengue vaccine.	No	[31]
(Sumile et al., 2020)	Vaccine	5	Composite score using 5 questions: perceived safety of vaccines; perceived effectiveness and necessity of vaccines; acceptance of the selection and scheduling of vaccines; positive values and affect toward vaccines; and perceived legitimacy of authorities to require vaccinations.	No	[32]
(Hadisoemarto and Castro 2013)	Vaccine	1	Would it be likely to vaccine your child.	NA	[33]
(Rodriguez et al., 2023)	Vaccine	1	Willingness to receive tablet.	NA	[34]
(Curren et al., 2023)	Vaccine	1	Interested in vaccine.	NA	[35]
**Soil-transmitted helminth**					
(Perez et al., 2021)	Tablet	9	Composite score using 9 questions: result of the intake, patient reaction, preparation and administration time, divided dose, food/drink, reward, restraint, alteration, and device not provided.	Yes	[36]
(Palmeirim et al., 2020)	Tablet	6-7	6-7 questions analyzed independently: have you ever taken a tablet for belly worm, if yes, how did you eat it, do you like swallowing a pill whole with water, did you like the taste of the tablets, was the tablet too big, too small or good, would it be alright for you take this tablet again, do you prefer to chew or swallow the tablet.	No	[37]
(Bartkett et al., 2023)^%^	Tablet	19	19 questions analyzed independently: purpose of school preventative chemotherapy (PC), took PC at school last year, reason for taking PC, easy to take medicine, side effects to medicine, specific side effect to medicine, treatment for side effects, why they did not take medicines, agree with need to control soil-transmitted helminth (STH) infection, agree with need to control schistosomiasis (SCH) infection, willing to take STH medicine in future, reason for willingness to take STH in future, reason for not willing to take STH medicine future, preference for delivery of STH medicine in future, willing to take SCH medicine in future, reason for willingness to take SCH medicine in future, reason for not willing to take SCH medicine future, preference for delivery SCH medicine in future, happy with delivery of PC at school, happy with teachers delivering PC.	No	[38]
(Parikh et al., 2012)	Tablet	4	4 questions analyzed independently: MDA would be more efficient if teachers administer medication, teachers should be able to give medication to students, teachers would be able to administer medications safely, students would be willingly to take deworming tablets.	No	[39]
**Onchocerciasis**					
(Kumah et al., 2023)	Tablet	9*	Composite score using 9 questions: this drugs works against onchocerciasis or river blindness, this drug reduces the burden of itching, this drug works against the occurrence and severity of skin symptoms, I would take this treatment again, I would recommend this treatment to my relatives, I would be willing to change my family’s routine so that we take the treatment again, the benefits of this treatment outweigh its side effects, this treatment is a good way to help our health problems here, overall, mass drug administration of ivermectin will help my community.	Yes	[40]
(Pacque et al., 1991)	Tablet	1	Received treatment.	NA	[41]
(Pacque et al., 1990)	Tablet	1	Received treatment.	NA	[42]
**Scabies**					
(Abdel-Raheem et al., 2016)	Tablet	1	Experienced side effects.	NA	[43]
(Alebiosu et al., 2003)	Ointment	NA	The authors state that a questionnaire was administered to each patient to obtain information on the acceptability of the ointment and soap (appearance, smell, dosing and method of administration. Then report that “the acceptability of the ointment and soap in terms of appearance, smell, frequency of dosing, and method of application was 100%”. No other information is provided.	NA	[44]
**Leishmaniasis**					
(Khabsa et al., 2022)	Tablet	1	Is combination therapy more acceptable.	NA	[45]
(Mondal et al., 2014)	Tablet	1	Consented to treatment.	NA	[46]
**Schistosomiasis**					
(Diop et al., 2025)	Tablet	1	Received treatment.	NA	[47]
(Kimani et al., 2018)	Tablet	1	Spat and/or vomited treatment.	NA	[48]
(Edwards et al, 2025)	Table	6	Composite score using 6 questions: Is schistosomiasis drugs pleasing to you, do you have any objections to take schistosomiasis drugs, do you like the schistosomiasis drugs (praziquantel), are you okay with taking schistosomiasis drugs (praziquantel), according to you, is schistosomiasis drugs (Praziquantel) a good medication/intervention, is this schistosomiasis drug a satisfactory medication/intervention to you.	Yes	[49]
**Trachoma**					
(Desmond et al., 2005)	Tablet	1	Received treatment.	NA	[50]
**Dracunculiasis**					
(Sastry et al., 1978)	Tablet	NA	The authors state that “the patients accepted the drug readily because of its agreeable taste”. No other information is provided.	NA	[51]

* This instrument is utilized across multiple studies.

& Article also investigates soil-transmitted helminths.

% Article also investigates schistosomiasis.

### Articles that measure acceptability with multiple questions and with a defined threshold for acceptability

Nine articles measure acceptability using multiple questions and a defined threshold for acceptability (Table 4). Five articles measured acceptability using a composite score developed by Krentel et al. (2021), which sums the values of nine acceptability indicators based on a four-point scale – disagree a lot, disagree, agree, agree a lot [10–12,26,40]. The median score of 22.5 (range: 9–36) was used as the threshold for acceptability. The measure was adapted from the Interventions Rating Tool [52] where the median threshold was also determined. Edwards et al. (2025) used a similar methodology to measure acceptability using a composite score adapted from the Theoretical Framework for Acceptability [4], which sums the value of six acceptability indicators based on a five-point scale – strongly disagree, disagree, no opinion, agree, strongly agree. This metric uses the median (50^th^ percentile) as the acceptability threshold (high versus low acceptability). Perez et al. (2021) measured acceptability using the ClinSearch Acceptability Score Test (CAST) [53], performing a multiple correspondence analysis of nine behaviors, each based on two to three response options [36]. For this approach, the results were plotted on a 3D map with confidence ellipses, and acceptability was determined by whether the barycenter and confidence ellipses fell within a specific area of the map. The other two articles evaluated acceptability for each specific question rather than creating a composite score. Nujum et al. (2012) assessed acceptability using four questions based on a four-point scale [27]. The median for each question (range and median not indicated in the article) was used as the threshold for acceptability. Arham et al. (2022) assessed acceptability using seven questions based on a seven-point scale [30]. They categorized each acceptability question as follows: scores between 1.00 and 3.00 were considered low, scores between 3.01 and 5.00 as moderate, and scores between 5.01 and 7.00 as high.

**Table 4:** Methods for measuring acceptability with multiple questions and with a defined threshold for acceptability.

Instrument and method used to measure acceptability	Threshold for acceptability	Citation
Acceptability was measured using a composite score developed by Krentel et al. (2021) which sums the values of 9 acceptability indicators: • These drugs work against lymphatic filariasis • These drugs work against itching • These drugs work against intestinal worms • I would take this treatment again • I would recommend this treatment to my relatives • I would be willing to change my family’s routine so that we take the treatment again • I liked this treatment, this treatment is a good way to help our health problems here, • Overall, this treatment will help my community The acceptability scores can range from 9 to 36, based on a four-point scale (1 = disagree a lot, 2 = disagree, 3 = agree, and 4 = agree a lot) for all nine questions. Kumah et al. (2023) adapted this scale to assess acceptability for onchocerciasis.	Two studies used the composite acceptability score [10,11], while three studies applied a threshold of 22.5 (the median score) to categorize responses as above or below the acceptability threshold [12,26,40].	[10–12,26,40]
Acceptability was measured using a composite score adapted from the Theoretical Framework for Acceptability [4], which sums the values of 6 acceptability indicators: • Is schistosomiasis drugs pleasing to you • Do you have any objections to take schistosomiasis drugs • Do you like the schistosomiasis drugs (praziquantel) • Are you okay with taking schistosomiasis drugs (praziquantel) • According to you, is schistosomiasis drugs (Praziquantel) a good medication/intervention • Is this schistosomiasis drug a satisfactory medication/intervention to you. The acceptability scores can range from 6 to 3, based on a five-point scale (1 = strongly disagree, 2 = disagree, 3 = no opinion, 4 = agree, 5 = strongly agree) for all six questions.	The variable was dichotomized as low acceptability for participants with scores below the median (50th percentile) and high acceptability for those with scores equal to or above the median.	[49]
Acceptability was measured using the ClinSearch Acceptability Score Test (CAST). This tool collects information on 9 observed behaviours including: 1. Result of the intake 2. Patient reaction 3. Preparation and administration time 4. Divided dose 5. Food/drink 6. Reward 7. Restraint 8. Alteration 9. Device not provided Each observed behaviour had 2–3 response options. A multiple correspondence analysis (MCA) was used to summarize the key information, with confidence ellipses around the barycenter for all dimension pairs indicating the likely position, which was then plotted on a 3D map.	If the barycenter, along with the entire confidence ellipse surrounding it, falls within the green area of the map, the medicine was classified as positively accepted.	[36]
Acceptability was measured in terms of 4 variables: 1. Sight 2. Cover 3. Number 4. Size. The acceptability score was based on a four-point scale. Specific questions and response options were not provided.	Using the median, all the acceptability related variables were dichotomized as low and high acceptability.	[27]
Acceptability was measured in terms of 7 variables based on previously published work by Amin and Hashim [54] and previous studies [55–63] including: 1. Trust in key players 2. Attitudes to technology 3. Religiosity 4. Perceived benefit 5. Perceived risk 6. Attitude to dengue vaccine. 7. Intention to dengue vaccine Respondents were asked to evaluate their opinion on a 7-point Likert scale ranging from 1 (strongly disagree) to 7 (strongly agree) for each question.	The acceptability threshold was defined as follows: scores between 1.00 and 3.00 were categorized as low, scores between 3.01 and 5.00 as moderate, and scores between 5.01 and 7.00 as high for each variable.	[30]

### Articles that measure acceptability with multiple questions and without a defined thresholds for acceptability

Five articles measure acceptability using multiple questions, but without a defined threshold for acceptability (Table 5). Sumile et al. (2020) measured acceptability through the agreement or disagreement of five questions [32]. The authors do not provide a methodology on deriving an acceptability score but report a mean vaccine acceptability score of 5.28. McMahon et al. (2019) assessed acceptability using nine questions and report the number of respondents who agreed with each statement, though the other possible response categories were not specified [31]. Palmeirim et al. (2020) used six to seven questions with varying response options (e.g., yes/no, chewed/swallowed) and report the number of respondents in each category [37]. Bartlett et al. (2023) measured acceptability through nineteen questions with response categories such as yes/no, school/central point/sanitary unit/other, and report the number of respondents for each option [38]. Parikh et al. (2013) assessed acceptability through the agreement or disagreement of four questions and also report the number of respondents for each category [39].

**Table 5:** Methods for measuring acceptability with multiple questions and without a defined threshold for acceptability.

Instrument and method used to measure acceptability	Citation
The authors adapted the Vaccine Acceptability Scale [64], which measures acceptability through the agreement or disagreement of 5 areas including: perceived safety of vaccines; perceived effectiveness and necessity of vaccines; acceptance of the selection and scheduling of vaccines; positive values and affect toward vaccines; and perceived legitimacy of authorities to require vaccinations. The methodology deriving the acceptability score was not provided by the authors, but they report a mean vaccine acceptability of 5.28.	[32]
Acceptability was measured in terms of 9 variables: vaccines are beneficial in improving health, vaccines are safe, a dengue vaccine would be useful in your community/country, you would get a dengue vaccine, you would vaccinate your child(ren) with the dengue vaccine, you would get vaccinated if protection from illness was for less than two years, dengue vaccines should be a standard vaccination, there will be political opposition to the dengue vaccine’s implementation, you would be willing to pay for the dengue vaccine. The authors report the number of respondents that agree with each statement, but the other possible response categories are not specified.	[31]
Acceptability was measured in terms of 6 or 7 variable depending treatment allocation: have you ever taken a tablet for belly worm, if yes, how did you eat it, do you like swallowing a pill whole with water, did you like the taste of the tablets, was the tablet too big, too small or good, would it be alright for you take this tablet again, do you prefer to chew or swallow the tablet. Each question had a range of responses.	[37]
Acceptability was measured in terms of 19 variables: purpose of school preventative chemotherapy (PC), took PC at school last year, reason for taking PC, easy to take medicine, side effects to medicine, specific side effect to medicine, treatment for side effects, why did not take medicines, agree with need to control soil-transmitted helminth (STH) infection, agree with need to control schistosomiasis (SCH) infection, willing to take STH medicine in future, reason for willingness to take STH in future, reason for not willing to take STH medicine future, preference for delivery of STH medicine in future, willing to take SCH medicine in future, reason for willingness to take SCH medicine in future, reason for not willing to take SCH medicine future, preference for delivery SCH medicine in future, happy with delivery of PC at school, happy with teachers delivering PC. Each question had a range of responses.	[38]
Acceptability was measured in terms of 4 variables: MDA would be more efficient if teachers administer medication, teachers should be able to give medication to students, teachers would be able to administer medications safely, students would be willingly to take deworming tablets. Respondents were asked to either agree, disagree, or state that they had no opinion.	[39]

### Articles that measure acceptability with a single question

Most articles in this scoping review (22/40, 55%) assessed acceptability using a single question. Eleven articles evaluated acceptability based on whether respondents chose to receive the treatment [17–21,25,41,42,47,50], while two articles focused on whether respondents chose to ingest the treatment [13,28] (Table 6). The remaining nine articles used binary or ordinal measures, ranging from anticipated experience with the intervention (i.e., willingness to receive the tablet [22]) and experienced response to the intervention (i.e., whether the respondent experienced side effects [43]).

**Table 6:** Methods for measuring acceptability with a single question.

Instrument and method used to measure acceptability	Citation
Acceptability was measured based on whether the respondent chose to receive the treatment.	[16–20,24,40,41,46,49]
Acceptability was measured based on whether the respondent chose to ingest the treatment.	[13,28]
Acceptability was measured based on whether the respondent spat and/or vomited all or part of the treatment.	[48]
Acceptability was measured in terms of proportion of patients who consented to treatment.	[46]
Acceptability was measured based on whether the respondent experienced side effects.	[43]
Acceptability was measured based on a hypothetical chikungunya vaccine and asked: if there was a vaccine to prevent chikungunya, would you be interested in it.	[35]
Vaccine acceptability was assessed under three hypothetical scenarios by asking “Would you get vaccinated if the vaccine was…” • recommended by health authorities and free • offered within a clinical trial • officially recommended but not covered, with an estimated cost corresponding to the actual retail price at pharmacies at the time of the study The acceptability classification was based on a five-point scale ranging from “yes, certainly, yes probably, I do not know yet, probably not, or certainly not”. Vaccine acceptability was subsequently recoded as a binary variable by grouping “yes certainly and yes probably”.	[29]
Acceptability was measured based on willingness to receive prophylaxis (tablets). The acceptability classification was based on a four-point scale: 1) acceptable are those that responded definitely and probably, and 2) not acceptable are those that responded probably not and definitely not.	[22]
Acceptability was measured based on intention to take a hypothetical vaccine: “if there was an approved vaccine for dengue, available free of cost, would you get it”. Dengue vaccine intention was considered as a binary response, where “no” or “unsure” values were grouped as “no”.	[34]
Acceptability was measured based on willingness of parents to vaccinate their child through the question: would it be likely for you to vaccinate your children. The acceptability classification was based on a five-point scale ranging from “very unlikely” to “very likely”.	[33]
Acceptability was measured based on a previously published question [65–68]: is combination therapy (as compared to monotherapy) more acceptable for the health care provider. Respondents were asked to respond with one of the following options: not acceptable, probably not acceptable, probably yes acceptable, yes acceptable, don’t know.	[45]

### Articles that report that they measure acceptability without an explanation of their methodology

Four articles report that they measured acceptability, but do not provide an explanation of their methodology (Table 7). For example, in Alebiosu et al. (2003), the authors state that they administered a questionnaire to each patient then report that “the acceptability of the ointment and soap in terms of appearance, smell, frequency of dosing, and method of application was 100%” [44]. However, there is no further information regarding the specific questions asked and the response options used, making it unclear how they arrived at the 100% figure.

**Table 7:** Methods for measuring acceptability is unclear.

Instrument and method used to measure acceptability	Citation
Acceptability of the regimens was determined by the severity of side effects and patient compliance with prescribed drugs. No other information is provided.	[23]
The authors state that “the patients accepted the drug readily because of its agreeable taste”. No other information is provided.	[51]
The authors state that a questionnaire was administered to each patient to obtain information on the acceptability of the ointment and soap. Then report that “the acceptability of the ointment and soap in terms of appearance, smell, frequency of dosing, and method of application was 100%”. No other information is provided.	[44]
The authors state that they had a questionnaire on knowledge of leprosy and acceptability of prophylaxis, then report that “an encouraging result was the finding that most respondents would accept chemoprophylaxis against leprosy being offered to family members”. No other information is provided.	[24]

## Discussion

This scoping review aimed to review how the acceptability of pharmaceutical interventions for the prevention and treatment of NTDs have been quantitatively measured in the peer-reviewed literature, with the goal of advancing toward a standardized approach for measuring acceptability. Our search yielded 1340 articles, of which 40 met the inclusion criteria. Notably, most of the included studies (20/40) were published within the last five years (2020–2025), with only 10 of the 21 NTDs having studies that quantitatively assessed the acceptability of an intervention. The methodologies used to measure acceptability in the literature varied widely: 14 articles employed multiple questions, with 9 explicitly defining a threshold for acceptability, while 22 relied on a single-question approach. Four articles reported assessing acceptability but did not provide methodological details. These results emphasize inconsistencies in how acceptability is measured and reported across studies.

These inconsistencies are particularly evident in the context of MDA, which is a key public health strategy designed to treat and prevent the spread of five NTDs classified as preventive chemotherapy NTDs (PC-NTDs). PC-NTDs, including LF, onchocerciasis, schistosomiasis, STH, and trachoma, are a group of diseases for which preventive treatment is recommended at the population level rather than targeting known cases. This approach involves distributing medications to entire at-risk populations, regardless of individual infection status, to reduce disease prevalence and to interrupt transmission. In this scoping review, all five PC-NTDs had published articles investigating the acceptability of one or more tablets, including lymphatic filariasis, soil-transmitted helminths, onchocerciasis, schistosomiasis, and trachoma. Although each PC-NTD requires a specific treatment approach - such as albendazole, diethylcarbamazine, and ivermectin (in various combinations depending on co-endemicity with loa loa and onchocerciasis) for lymphatic filariasis; albendazole for soil-transmitted helminths; ivermectin for onchocerciasis; praziquantel for schistosomiasis; and azithromycin for trachoma - the overarching strategy for implementing these programs remains largely consistent across diseases [9]. Despite the use of similar implementation approaches, the 16 articles focused on PC-NTDs included in this review employed 9 different methods to measure acceptability.

One of the most significant findings of this scoping review was the clear need for a standardized methodology to assess acceptability. This is particularly critical given that acceptability is one of the seven key considerations identified by the WHO when developing public health, health system, and policy recommendations. The *WHO Handbook for Guideline Development* (2nd edition) notes that “the greater the acceptability of an option to all or most stakeholders, the greater the likelihood of a strong recommendation” [1]. However, no specific guidance is provided on how to measure acceptability. Establishing a standardized methodology would support a more consistent and systematic approach to evaluating this important dimension.

Similarly, nearly a third of the articles (13/40) stated that they measured acceptability, however their methodology revealed that they were measuring coverage (received treatment) and compliance (swallowed treatment). This aligns with observations in two systematic reviews by Shuford et al. (2016) and Maddren et al. (2023), that urge the need for standardized terminology to differentiate between coverage and compliance parameters [69–71]. Misclassifying these parameters not only limits the comparability of findings across studies, but also limits efforts to understand and improve acceptability of NTD interventions, which plays a critical role in the uptake and success of interventions.

It is worth noting that acceptability was often measured using a single question (22/40 articles), despite it being widely recognized as a multi-faceted construct [4]. Capturing the complexity of a multi-faceted construct with just one question raises significant concerns about the depth and reliability of these assessments. Furthermore, only 9 studies established a clear threshold for what constitutes acceptability. Without defining a tipping point, it becomes challenging to determine whether an intervention is truly deemed acceptable or not. The lack of transparency and replicability in four of the articles further complicates efforts to develop standardized methodologies, as it remains unclear how many of these responses were initially derived.

These methodological inconsistencies are compounded by an even larger gap in the research where more than half of the 21 NTDs have no studies quantitatively assessing acceptability, despite the existence of efficacious pharmaceutical interventions. For instance, the WHO recommends an 8-week regimen of specific antibiotics for Buruli ulcer [72], and benznidazole or nifurtimox, both of which have high cure rates, for chagas disease [73]. Yet, no published studies were identified that quantitatively evaluate the acceptability of these treatments.

### Limitations

This study was subject to some limitations. Nearly all studies included in this review used a different methodology to measure the acceptability of a pharmaceutical intervention for an NTD. This lack of consistency makes it challenging to compare findings across studies and challenges the progress toward developing a standardized methodology.

### Conclusion

This scoping review highlights significant gaps and inconsistencies in the quantitative measurement of acceptability for pharmaceutical interventions for NTDs. The lack of standardized methodologies hinders the ability to compare findings across studies, establish benchmarks, and draw actionable conclusions – ultimately limiting the utility of acceptability in the guideline development process. Furthermore, the absence of quantitative studies for over half of the NTDs underscores the need to prioritize research in this area. To improve the effectiveness of interventions and support global NTD elimination efforts, future work must focus on developing and adopting standardized, transparent frameworks for measuring acceptability. Addressing these gaps is essential for advancing equitable and effective healthcare delivery to populations most in need.

## Supporting information

S1 File PRISMA-ScR Checklist. From: Tricco AC, Lillie E, Zarin W, O’Brien KK, Colquhoun H, Levac D, et al. PRISMA Extension for Scoping Reviews (PRISMA-ScR): Checklist and Explanation. Ann Intern Med. 2018;169:467–473. https://doi.org/10.7326/M18-0850. This material is licensed under a CC BY 4.0 license.
(DOCX)

S2 File Search strategy. (DOCX)
