# Supplementary material for: Approaches to quantifying acceptability of pharmaceutical interventions for neglected tropical diseases: A scoping review
Source: PLoS Negl Trop Dis. 2026 May 18;20(5):e0014272. doi: 10.1371/journal.pntd.0014272 (PMC13183195; doi:10.1371/journal.pntd.0014272)
Supplement: S2 File — (DOCX) [file pntd.0014272.s002.docx]

**Ovid MEDLINE (598)**

Ovid MEDLINE(R) ALL <1946 to November 18, 2025>

| 1 | Neglected Diseases/ | 2850 |
| --- | --- | --- |
| 2 | (neglect* adj4 disease*).ti,ab,kf. | 12647 |
| 3 | Buruli Ulcer/ | 786 |
| 4 | ((buruli* or bairnsdale or daintree or searls) adj4 (ulcer* or disease*)).ti,ab,kf. | 1310 |
| 5 | Mycobacterium ulcerans/ | 951 |
| 6 | (Mycobacteri* adj4 (ulcer* or infect* or buruli*)).ti,ab,kf. | 27956 |
| 7 | Chagas Disease/ | 13987 |
| 8 | (chagas* adj4 disease*).ti,ab,kf. | 15755 |
| 9 | (american adj4 trypanosom*).ti,ab,kf. | 1619 |
| 10 | Trypanosoma cruzi/ | 14089 |
| 11 | ((Trypanosom* or Schizotrypanum) adj4 cruz*).ti,ab,kf. | 17341 |
| 12 | exp Dengue/ or Dengue Virus/ | 21705 |
| 13 | ((breakbone* or break-bone* or dandy or denque or aden or bouquet or solar or sun) adj4 (fever* or classical or virus* or disease* or infect* or flavivirus*)).ti,ab,kf. | 645 |
| 14 | (DENV or dengue*).ti,ab,kf. | 33155 |
| 15 | Chikungunya virus/ or Chikungunya Fever/ | 5094 |
| 16 | (CHIKUNGUNYA or CHIcKUNGUNYA or CHKV).ti,ab,kf. | 8698 |
| 17 | Dracunculiasis/ or Dracunculus Nematode/ | 967 |
| 18 | ((guinea or medina or gorstius or dragon or serpent) adj4 (worm* or medinens*)).ti,ab,kf. | 532 |
| 19 | (dracuncul* or dracontiasis or guineaworm).ti,ab,kf. | 1305 |
| 20 | exp Echinococcosis/ or exp Echinococcus/ | 22560 |
| 21 | (echinococc* or hydatid*).ti,ab,kf. | 30094 |
| 22 | clonorchiasis/ or fascioliasis/ or opisthorchiasis/ or paragonimiasis/ | 8259 |
| 23 | CLONORCHIS SINENSIS/ or Opisthorchis/ or exp Fasciola/ or exp Paragonimus/ | 7825 |
| 24 | (clonorchi* or opisthorchi* or Fasciol* or Paragonim*).ti,ab,kf. | 13455 |
| 25 | (lung adj2 fluke*).ti,ab,kf. | 328 |
| 26 | exp Trypanosoma brucei gambiense/ or exp Trypanosoma brucei rhodesiense/ | 1912 |
| 27 | exp Trypanosomiasis, African/ | 6248 |
| 28 | ((sleeping or african or negro or gambian or rhodesian or congo) adj4 (sickness* or letharg* or trypanosom*)).ti,ab,kf. | 8098 |
| 29 | (trypanosom* adj4 (brucei or infect* or gambiense* or rhodesiense*)).ti,ab,kf. | 17829 |
| 30 | (nelavan or nagana).ti,ab,kf. | 317 |
| 31 | exp Leishmaniasis/ or exp Leishmania/ | 36996 |
| 32 | leishmania*.ti,ab,kf. | 43299 |
| 33 | exp leprosy/ or Mycobacterium leprae/ | 25288 |
| 34 | ((hansen* or lepra* or lepro* or icrc) adj4 (disease* or graecorum or infect* or morbus or bacill* or mycoblasma)).ti,ab,kf. | 5718 |
| 35 | (lepro* or hansenias*).ti,ab,kf. | 26433 |
| 36 | Elephantiasis, Filarial/ or WUCHERERIA BANCROFTI/ or BRUGIA MALAYI/ | 5075 |
| 37 | elephantias*.ti,ab,kf. | 1779 |
| 38 | ((wuchereria or filaria* or wucheria or microfilaria* or brugia or brugoa) adj4 (lymph* or bancrofti* or brugian or malay* or bancrofti or sanguinis or timori)).ti,ab,kf. | 7220 |
| 39 | Mycetoma/ or Chromoblastomycosis/ | 3331 |
| 40 | ((madura or dermatitis or mossy or pedrosoi or phialophora) adj4 (foot or feet or verruc* or fonsecaea)).ti,ab,kf. | 1056 |
| 41 | (actinomycetoma* or maduromycos* or eumycetoma* or mycetoma* or chromoblastomycos* or chromomycos*).ti,ab,kf. | 3791 |
| 42 | Noma/ | 579 |
| 43 | (noma or nomas).ti,ab,kf. | 1030 |
| 44 | ((cancrum or stomatiti* or mouth or oral) adj4 (oris or gangrenous or denture or ulcer* or epitheli* or mucosa or recurrent or granulomatosa or virus*)).ti,ab,kf. | 50671 |
| 45 | exp Onchocerciasis/ or Onchocerca volvulus/ | 4677 |
| 46 | (volvulos* or oncho*).ti,ab,kf. | 6659 |
| 47 | (river adj4 blindness).ti,ab,kf. | 458 |
| 48 | rabies/ or rabies virus/ | 13275 |
| 49 | (rabies or raby or hydrophobia or lyssa* or hubert or rabbia).ti,ab,kf. | 18144 |
| 50 | scabies/ or Sarcoptes scabiei/ | 4209 |
| 51 | scabie*.ti,ab,kf. | 5387 |
| 52 | ((watchmaker or sarcopt* or seven) adj4 (mange or scabi* or itch*)).ti,ab,kf. | 1491 |
| 53 | schistosomasis/ or Schistosomiasis haematobia/ or Schistosomiasis japonica/ or Schistosomiasis mansoni/ or Schistosoma haematobium/ or Schistosoma japonicum/ or Schistosoma mansoni/ | 20882 |
| 54 | ((snail* or mason*) adj4 (fever* or disease*)).ti,ab,kf. | 235 |
| 55 | (bilharzia* or katayama or schistoma* or schistosom* or bilharzios* or schistomiasis or haematobi* or mansoni).ti,ab,kf. | 37717 |
| 56 | Ascariasis/ or Ascaris lumbricoides/ or Trichuriasis/ or TRICHURIS/ or Ancylostoma/ or Necator americanus/ | 9762 |
| 57 | (ascari* or roundworm* or lumbricoides or trichuri* or whipworm* or trichocephal* or hookworm* or ancylostoma* or necator*).ti,ab,kf. | 22932 |
| 58 | ((soil or transmitted) adj3 helminth*).ti,ab,kf. | 2852 |
| 59 | Snake Bites/ | 6106 |
| 60 | snakebite*.ti,ab,kf. | 4018 |
| 61 | (snake* adj4 (bite* or envenom*)).ti,ab,kf. | 4954 |
| 62 | Taeniasis/ or Taenia solium/ or cycticercosis/ or CYSTICERCUS/ | 4078 |
| 63 | (taenia* or cysticercos* or cysticercus or coenur* or cysticerciasis).ti,ab,kf. | 12544 |
| 64 | ((tapeworm* or solium*) adj4 (infect* or pork*)).ti,ab,kf. | 1948 |
| 65 | trachoma/ or CHLAMYDIA TRACHOMATIS/ | 17277 |
| 66 | trachoma*.ti,ab,kf. | 20506 |
| 67 | ((granular or Egyptian) adj4 (conjunctivitis or ophthalmia)).ti,ab,kf. | 32 |
| 68 | yaws/ or TREPONEMA PALLIDUM/ | 5576 |
| 69 | (frambesia* or yaw* or framboesia* or parangi or pian or pertenue or treponema or pallid*).ti,ab,kf. | 35961 |
| 70 | or/1-69 | 465330 |
| 71 | acceptabilit*.ti,ab,kf. | 67923 |
| 72 | 70 and 71 | 598 |

**Embase (998)**

Embase Classic+Embase <1947 to 2025 November 18>

| 1 | Neglected Diseases/ | 3099 |
| --- | --- | --- |
| 2 | (neglect* adj4 disease*).ti,ab,kf. | 15916 |
| 3 | Buruli Ulcer/ | 1544 |
| 4 | ((buruli* or bairnsdale or daintree or searls) adj4 (ulcer* or disease*)).ti,ab,kf. | 1579 |
| 5 | Mycobacterium ulcerans/ | 1598 |
| 6 | (Mycobacteri* adj4 (ulcer* or infect* or buruli*)).ti,ab,kf. | 35824 |
| 7 | exp Chagas Disease/ | 22250 |
| 8 | (chagas* adj4 disease*).ti,ab,kf. | 20050 |
| 9 | (american adj4 trypanosom*).ti,ab,kf. | 1420 |
| 10 | Trypanosoma cruzi/ | 20199 |
| 11 | ((Trypanosom* or Schizotrypanum) adj4 cruz*).ti,ab,kf. | 20119 |
| 12 | exp Dengue/ or exp Dengue Virus/ | 44739 |
| 13 | ((breakbone* or break-bone* or dandy or denque or aden or bouquet or solar or sun) adj4 (fever* or classical or virus* or disease* or infect* or flavivirus*)).ti,ab,kf. | 972 |
| 14 | (DENV or dengue*).ti,ab,kf. | 43388 |
| 15 | Chikungunya virus/ or Chikungunya Fever/ | 11559 |
| 16 | (CHIKUNGUNYA or CHIcKUNGUNYA or CHKV).ti,ab,kf. | 11216 |
| 17 | Dracunculiasis/ or Dracunculus medinensis/ | 1359 |
| 18 | ((guinea or medina or gorstius or dragon or serpent) adj4 (worm* or medinens*)).ti,ab,kf. | 570 |
| 19 | (dracuncul* or dracontiasis or guineaworm).ti,ab,kf. | 1612 |
| 20 | exp Echinococcosis/ or exp Echinococcus/ | 37082 |
| 21 | (echinococc* or hydatid*).ti,ab,kf. | 39528 |
| 22 | clonorchiasis/ or fascioliasis/ or opisthorchiasis/ or paragonimiasis/ | 10210 |
| 23 | CLONORCHIS SINENSIS/ or exp Opisthorchis/ or exp Fasciola/ or exp Paragonimus/ | 12236 |
| 24 | (clonorchi* or opisthorchi* or Fasciol* or Paragonim*).ti,ab,kf. | 15961 |
| 25 | (lung adj2 fluke*).ti,ab,kf. | 446 |
| 26 | exp Trypanosoma brucei gambiense/ or exp Trypanosoma brucei rhodesiense/ | 2399 |
| 27 | exp Trypanosomiasis, African/ | 6835 |
| 28 | ((sleeping or african or negro or gambian or rhodesian or congo) adj4 (sickness* or letharg* or trypanosom*)).ti,ab,kf. | 9175 |
| 29 | (trypanosom* adj4 (brucei or infect* or gambiense* or rhodesiense*)).ti,ab,kf. | 20257 |
| 30 | (nelavan or nagana).ti,ab,kf. | 379 |
| 31 | exp Leishmaniasis/ or exp Leishmania/ | 57427 |
| 32 | leishmania*.ti,ab,kf. | 51056 |
| 33 | exp leprosy/ or Mycobacterium leprae/ | 43149 |
| 34 | ((hansen* or lepra* or lepro* or icrc) adj4 (disease* or graecorum or infect* or morbus or bacill* or mycoblasma)).ti,ab,kf. | 8942 |
| 35 | (lepro* or hansenias*).ti,ab,kf. | 36802 |
| 36 | exp Lymphatic Filariasis/ or WUCHERERIA BANCROFTI/ or BRUGIA MALAYI/ | 8417 |
| 37 | elephantias*.ti,ab,kf. | 2529 |
| 38 | ((wuchereria or filaria* or wucheria or microfilaria* or brugia or brugoa) adj4 (lymph* or bancrofti* or brugian or malay* or bancrofti or sanguinis or timori)).ti,ab,kf. | 9289 |
| 39 | Mycetoma/ or chromomycosis/ | 5054 |
| 40 | ((madura or dermatitis or mossy or pedrosoi or phialophora) adj4 (foot or feet or verruc* or fonsecaea)).ti,ab,kf. | 1472 |
| 41 | (actinomycetoma* or maduromycos* or eumycetoma* or mycetoma* or chromoblastomycos* or chromomycos*).ti,ab,kf. | 5218 |
| 42 | stomatitis/ | 33351 |
| 43 | (noma or nomas).ti,ab,kf. | 2060 |
| 44 | ((cancrum or stomatiti* or mouth or oral) adj4 (oris or gangrenous or denture or ulcer* or epitheli* or mucosa or recurrent or granulomatosa or virus*)).ti,ab,kf. | 67085 |
| 45 | exp Onchocerciasis/ or Onchocerca volvulus/ | 8343 |
| 46 | (volvulos* or oncho*).ti,ab,kf. | 8177 |
| 47 | (river adj4 blindness).ti,ab,kf. | 588 |
| 48 | rabies/ or rabies virus/ | 21908 |
| 49 | (rabies or raby or hydrophobia or lyssa* or hubert or rabbia).ti,ab,kf. | 21914 |
| 50 | scabies/ or Sarcoptes scabiei/ | 9244 |
| 51 | scabie*.ti,ab,kf. | 7569 |
| 52 | ((watchmaker or sarcopt* or seven) adj4 (mange or scabi* or itch*)).ti,ab,kf. | 1897 |
| 53 | schistosomiasis/ or Schistosomiasis haematobia/ or Schistosomiasis japonica/ or Schistosomiasis mansoni/ or Schistosoma haematobium/ or Schistosoma japonicum/ or Schistosoma mansoni/ | 47007 |
| 54 | ((snail* or mason*) adj4 (fever* or disease*)).ti,ab,kf. | 285 |
| 55 | (bilharzia* or katayama or schistoma* or schistosom* or bilharzios* or schistomiasis or haematobi* or mansoni).ti,ab,kf. | 46544 |
| 56 | Ascariasis/ or Ascaris lumbricoides/ or Trichuriasis/ or TRICHURIS/ or ANCYLOSTOMATOIDEA/ or Ancylostoma duodenale/ or Necator americanus/ | 18653 |
| 57 | (ascari* or roundworm* or lumbricoides or trichuri* or whipworm* or trichocephal* or hookworm* or ancylostoma* or necator*).ti,ab,kf. | 28501 |
| 58 | ((soil or transmitted) adj3 helminth*).ti,ab,kf. | 3898 |
| 59 | Snakebite/ | 9993 |
| 60 | snakebite*.ti,ab,kf. | 5424 |
| 61 | (snake* adj4 (bite* or envenom*)).ti,ab,kf. | 6725 |
| 62 | Taenia solium infection/ or Taenia solium/ or cycticercosis/ or CYSTICERCUS/ | 6868 |
| 63 | (taenia* or cysticercos* or cysticercus or coenur* or cysticerciasis).ti,ab,kf. | 15389 |
| 64 | ((tapeworm* or solium*) adj4 (infect* or pork*)).ti,ab,kf. | 1833 |
| 65 | trachoma/ or CHLAMYDIA TRACHOMATIS/ | 29611 |
| 66 | trachoma*.ti,ab,kf. | 27251 |
| 67 | ((granular or Egyptian) adj4 (conjunctivitis or ophthalmia)).ti,ab,kf. | 19 |
| 68 | yaws/ or TREPONEMA PALLIDUM/ | 11836 |
| 69 | (frambesia* or yaw* or framboesia* or parangi or pian or pertenue or treponema or pallid*).ti,ab,kf. | 49208 |
| 70 | or/1-69 | 658738 |
| 71 | acceptabilit*.ti,ab,kf. | 96031 |
| 72 | 70 and 71 | 998 |

**CINAHL (129)**

| **#** | **Query** | **Results** |
| --- | --- | --- |
| S72 | S70 AND S71 | 129 |
| S71 | TI acceptabilit* OR AB acceptabilit* | 25,203 |
| S70 | S1 OR S2 OR S3 OR S4 OR S5 OR S6 OR S7 OR S8 OR S9 OR S10 OR S11 OR S12 OR S13 OR S14 OR S15 OR S16 OR S17 OR S18 OR S19 OR S20 OR S21 OR S22 OR S23 OR S24 OR S25 OR S26 OR S27 OR S28 OR S29 OR S30 OR S31 OR S32 OR S33 OR S34 OR S35 OR S36 OR S37 OR S38 OR S39 OR S40 OR S41 OR S42 OR S43 OR S44 OR S45 OR S46 OR S47 OR S48 OR S49 OR S50 OR S51 OR S52 OR S53 OR S54 OR S55 OR S56 OR S57 OR S58 OR S59 OR S60 OR S61 OR S62 OR S63 OR S64 OR S65 OR S66 OR S67 OR S68 OR S69 | 43,602 |
| S69 | TI ( frambesia* or yaw* or framboesia* or parangi or pian or pertenue or treponema or pallid* ) OR AB ( frambesia* or yaw* or framboesia* or parangi or pian or pertenue or treponema or pallid* ) | 3,491 |
| S68 | (MH "Yaws") | 59 |
| S67 | TI ( (granular or Egyptian) N4 (conjunctivitis or ophthalmia) ) OR AB ( (granular or Egyptian) N4 (conjunctivitis or ophthalmia) ) | 44 |
| S66 | TI trachoma* OR AB trachoma* | 3,175 |
| S65 | (MH "Chlamydia Trachomatis") | 2,042 |
| S64 | (MH "Trachoma") | 381 |
| S63 | TI ( (tapeworm* or solium*) N4 (infect* or pork*) ) OR AB ( (tapeworm* or solium*) N4 (infect* or pork*) ) | 105 |
| S62 | TI ( taenia* or cysticercos* or cysticercus or coenur* or cysticerciasis ) OR AB ( taenia* or cysticercos* or cysticercus or coenur* or cysticerciasis ) | 657 |
| S61 | (MH "Taeniasis") | 107 |
| S60 | TI ( snake* N4 (bite* or envenom*) ) OR AB ( snake* N4 (bite* or envenom*) ) | 662 |
| S59 | TI snakebite* OR AB snakebite* | 602 |
| S58 | (MH "Snake Bites") | 1,045 |
| S57 | TI ( (soil or transmitted) N3 helminth* ) OR AB ( (soil or transmitted) N3 helminth* ) | 369 |
| S56 | TI ( ascari* or roundworm* or lumbricoides or trichuri* or whipworm* or trichocephal* or hookworm* or ancylostoma* or necator ) OR AB ( ascari* or roundworm* or lumbricoides or trichuri* or whipworm* or trichocephal* or hookworm* or ancylostoma* or necator ) | 1,030 |
| S55 | (MH "Ascaris") OR (MH "Ascariasis") | 386 |
| S54 | TI ( bilharzia* or katayama or schistoma* or schistosom* or bilharzios* or schistomiasis or haematobi* or mansoni ) OR AB ( bilharzia* or katayama or schistoma* or schistosom* or bilharzios* or schistomiasis or haematobi* or mansoni ) | 1,606 |
| S53 | TI ( (snail* or mason*) N4 (fever* or disease*) ) OR AB ( (snail* or mason*) N4 (fever* or disease*) ) | 9 |
| S52 | (MH "Schistosomiasis") | 1,245 |
| S51 | TI ( (watchmaker or sarcopt* or seven) N4 (mange or scabi* or itch*) ) OR AB ( (watchmaker or sarcopt* or seven) N4 (mange or scabi* or itch*) ) | 132 |
| S50 | TI scabie* OR AB scabie* | 815 |
| S49 | (MH "Scabies") | 894 |
| S48 | TI ( rabies or raby or hydrophobia or lyssa* or hubert or rabbia ) OR AB ( rabies or raby or hydrophobia or lyssa* or hubert or rabbia ) | 1,575 |
| S47 | (MH "Rabies") | 1,366 |
| S46 | TI river N4 blindness OR AB river N4 blindness | 56 |
| S45 | TI ( volvulos* or oncho* ) OR AB ( volvulos* or oncho* ) | 338 |
| S44 | (MH "Onchocerciasis") | 212 |
| S43 | TI ( (cancrum or stomatiti* or mouth or oral) N4 (oris or gangrenous or denture or ulcer* or epitheli* or mucosa or recurrent or granulomatosa or virus*) ) OR AB ( (cancrum or stomatiti* or mouth or oral) N4 (oris or gangrenous or denture or ulcer* or epitheli* or mucosa or recurrent or granulomatosa or virus*) ) | 6,671 |
| S42 | TI ( noma or nomas ) OR AB ( noma or nomas ) | 173 |
| S41 | (MH "Stomatitis") | 2,248 |
| S40 | TI ( actinomycetoma* or maduromycos* or eumycetoma* or mycetoma* or chromoblastomycos* or chromomycos* ) OR AB ( actinomycetoma* or maduromycos* or eumycetoma* or mycetoma* or chromoblastomycos* or chromomycos* ) | 280 |
| S39 | TI ( (madura or dermatitis or mossy or pedrosoi or phialophora) N4 (foot or feet or verruc* or fonsecaea) ) OR AB ( (madura or dermatitis or mossy or pedrosoi or phialophora) N4 (foot or feet or verruc* or fonsecaea) ) | 90 |
| S38 | TI ( (wuchereria or filaria* or wucheria or microfilaria* or brugia or brugoa) N4 (lymph* or bancrofti* or brugian or malay* or bancrofti or sanguinis or timori) ) OR AB ( (wuchereria or filaria* or wucheria or microfilaria* or brugia or brugoa) N4 (lymph* or bancrofti* or brugian or malay* or bancrofti or sanguinis or timori) ) | 431 |
| S37 | TI elephantias* OR AB elephantias* | 133 |
| S36 | (MH "Elephantiasis, Filarial") | 320 |
| S35 | TI ( lepro* or hansenias* ) OR AB ( lepro* or hansenias* ) | 2,102 |
| S34 | TI ( (hansen* or lepra* or lepro* or icrc) N4 (disease* or graecorum or infect* or morbus or bacill* or mycoblasma) ) OR AB ( (hansen* or lepra* or lepro* or icrc) N4 (disease* or graecorum or infect* or morbus or bacill* or mycoblasma) ) | 594 |
| S33 | (MH "Hansen's Disease") | 1,837 |
| S32 | TI leishmania* OR AB leishmania* | 2,631 |
| S31 | (MH "Leishmania") | 769 |
| S30 | (MH "Leishmaniasis") | 1,950 |
| S29 | TI ( nelavan or nagana ) OR AB ( nelavan or nagana ) | 1 |
| S28 | TI ( trypanosom* N4 (brucei or infect* or gambiense* or rhodesiense*) ) OR AB ( trypanosom* N4 (brucei or infect* or gambiense* or rhodesiense*) ) | 458 |
| S27 | TI ( (sleeping or african or negro or gambian or rhodesian or congo) N4 (sickness* or letharg* or trypanosom*) ) OR AB ( (sleeping or african or negro or gambian or rhodesian or congo) N4 (sickness* or letharg* or trypanosom*) ) | 313 |
| S26 | (MH "Trypanosomiasis") | 1,329 |
| S25 | TI lung N2 fluke* OR AB lung N2 fluke* | 16 |
| S24 | TI ( clonorchi* or opisthorchi* or Fasciol* or Paragonim* ) OR AB ( clonorchi* or opisthorchi* or Fasciol* or Paragonim* ) | 366 |
| S23 | (MH "Paragonimiasis") | 53 |
| S22 | (MH "Opisthorchiasis") | 32 |
| S21 | (MH "Fascioliasis") | 132 |
| S20 | (MH "Clonorchiasis") | 33 |
| S19 | TI ( echinococc* or hydatid* ) OR AB ( echinococc* or hydatid* ) | 2,579 |
| S18 | (MH "Echinococcosis") OR (MH "Echinococcosis, Hepatic") | 1,634 |
| S17 | TI ( dracuncul* or dracontiasis or guineaworm ) OR AB ( dracuncul* or dracontiasis or guineaworm ) | 292 |
| S16 | TI ( (guinea or medina or gorstius or dragon or serpent) N4 (worm* or medinens*) ) OR AB ( (guinea or medina or gorstius or dragon or serpent) N4 (worm* or medinens*) ) | 74 |
| S15 | (MH "Dracunculiasis") | 249 |
| S14 | TI ( CHIKUNGUNYA or CHIcKUNGUNYA or CHKV ) OR AB ( CHIKUNGUNYA or CHIcKUNGUNYA or CHKV ) | 1,220 |
| S13 | (MH "Chikungunya Fever") OR (MH "Chikungunya Virus") | 782 |
| S12 | TI ( DENV or dengue ) OR AB ( DENV or dengue ) | 4,372 |
| S11 | TI ( (breakbone* or break-bone* or dandy or denque or aden or bouquet or solar or sun) N4 (fever* or classical or virus* or disease* or infect* or flavivirus*) ) OR AB ( (breakbone* or break-bone* or dandy or denque or aden or bouquet or solar or sun) N4 (fever* or classical or virus* or disease* or infect* or flavivirus*) ) | 121 |
| S10 | (MH "Dengue") | 3,068 |
| S9 | TI ((Trypanosom or Schizotrypanum) N4 cruz* ) OR AB ( (Trypanosoma or Schizotrypanum) N4 cruz* ) | 437 |
| S8 | TI american N4 trypanosom* OR AB american N4 trypanosomias* | 52 |
| S7 | TI chagas* N4 disease* OR AB chagas* N4 disease* | 1,087 |
| S6 | (MH "Trypanosomiasis") | 1,329 |
| S5 | TI (Mycobacteri* N4 (ulcer* or infection* or buruli*) ) OR AB ( Mycobacteri* N4 (ulcer*or infect* or buruli*) ) | 1,384 |
| S4 | TI ( (buruli* or bairnsdale or daintree or searls) N4 (ulcer* or disease*) ) OR AB ( (buruli* or bairnsdale or daintree or searls) N4 (ulcer* or disease*) ) | 172 |
| S3 | (MH "Buruli Ulcer") | 88 |
| S2 | TI neglect* N4 disease* OR AB neglect* N4 disease* | 1,458 |
| S1 | (MH "Neglected Diseases") | 169 |

**Global health (423)**

| **#** | **Query** | **Results** |
| --- | --- | --- |
| S72 | S70 AND S71 | 423 |
| S71 | TI acceptabilit* OR AB acceptabilit* | 25,210 |
| S70 | S1 OR S2 OR S3 OR S4 OR S5 OR S6 OR S7 OR S8 OR S9 OR S10 OR S11 OR S12 OR S13 OR S14 OR S15 OR S16 OR S17 OR S18 OR S19 OR S20 OR S21 OR S22 OR S23 OR S24 OR S25 OR S26 OR S27 OR S28 OR S29 OR S30 OR S31 OR S32 OR S33 OR S34 OR S35 OR S36 OR S37 OR S38 OR S39 OR S40 OR S41 OR S42 OR S43 OR S44 OR S45 OR S46 OR S47 OR S48 OR S49 OR S50 OR S51 OR S52 OR S53 OR S54 OR S55 OR S56 OR S57 OR S58 OR S59 OR S60 OR S61 OR S62 OR S63 OR S64 OR S65 OR S66 OR S67 OR S68 OR S69 | 268,549 |
| S69 | TI ( frambesia* or yaw* or framboesia* or parangi or pian or pertenue or treponema or pallid* ) OR AB ( frambesia* or yaw* or framboesia* or parangi or pian or pertenue or treponema or pallid* ) | 6,490 |
| S68 | (MH "Yaws") | 323 |
| S67 | TI ( (granular or Egyptian) N4 (conjunctivitis or ophthalmia) ) OR AB ( (granular or Egyptian) N4 (conjunctivitis or ophthalmia) ) | 3 |
| S66 | TI trachoma* OR AB trachoma* | 8,883 |
| S65 | (MH "Chlamydia Trachomatis") | 42 |
| S64 | (MH "Trachoma") | 2,276 |
| S63 | TI ( (tapeworm* or solium*) N4 (infect* or pork*) ) OR AB ( (tapeworm* or solium*) N4 (infect* or pork*) ) | 1,246 |
| S62 | TI ( taenia* or cysticercos* or cysticercus or coenur* or cysticerciasis ) OR AB ( taenia* or cysticercos* or cysticercus or coenur* or cysticerciasis ) | 10,876 |
| S61 | (MH "Taeniasis") | 2,560 |
| S60 | TI ( snake* N4 (bite* or envenom*) ) OR AB ( snake* N4 (bite* or envenom*) ) | 3,255 |
| S59 | TI snakebite* OR AB snakebite* | 2,668 |
| S58 | (MH "Snake Bites") | 3,667 |
| S57 | TI ( (soil or transmitted) N3 helminth* ) OR AB ( (soil or transmitted) N3 helminth* ) | 2,733 |
| S56 | TI ( ascari* or roundworm* or lumbricoides or trichuri* or whipworm* or trichocephal* or hookworm* or ancylostoma* or necator ) OR AB ( ascari* or roundworm* or lumbricoides or trichuri* or whipworm* or trichocephal* or hookworm* or ancylostoma* or necator ) | 18,774 |
| S55 | (MH "Ascaris") OR (MH "Ascariasis") | 4,238 |
| S54 | TI ( bilharzia* or katayama or schistoma* or schistosom* or bilharzios* or schistomiasis or haematobi* or mansoni ) OR AB ( bilharzia* or katayama or schistoma* or schistosom* or bilharzios* or schistomiasis or haematobi* or mansoni ) | 37,646 |
| S53 | TI ( (snail* or mason*) N4 (fever* or disease*) ) OR AB ( (snail* or mason*) N4 (fever* or disease*) ) | 265 |
| S52 | (MH "Schistosomiasis") | 32,962 |
| S51 | TI ( (watchmaker or sarcopt* or seven) N4 (mange or scabi* or itch*) ) OR AB ( (watchmaker or sarcopt* or seven) N4 (mange or scabi* or itch*) ) | 1,510 |
| S50 | TI scabie* OR AB scabie* | 3,252 |
| S49 | (MH "Scabies") | 2,353 |
| S48 | TI ( rabies or raby or hydrophobia or lyssa* or hubert or rabbia ) OR AB ( rabies or raby or hydrophobia or lyssa* or hubert or rabbia ) | 13,958 |
| S47 | (MH "Rabies") | 13,005 |
| S46 | TI river N4 blindness OR AB river N4 blindness | 382 |
| S45 | TI ( volvulos* or oncho* ) OR AB ( volvulos* or oncho* ) | 6,769 |
| S44 | (MH "Onchocerciasis") | 5,228 |
| S43 | TI ( (cancrum or stomatiti* or mouth or oral) N4 (oris or gangrenous or denture or ulcer* or epitheli* or mucosa or recurrent or granulomatosa or virus*) ) OR AB ( (cancrum or stomatiti* or mouth or oral) N4 (oris or gangrenous or denture or ulcer* or epitheli* or mucosa or recurrent or granulomatosa or virus*) ) | 8,476 |
| S42 | TI ( noma or nomas ) OR AB ( noma or nomas ) | 174 |
| S41 | (MH "Stomatitis") | 2,064 |
| S40 | TI ( actinomycetoma* or maduromycos* or eumycetoma* or mycetoma* or chromoblastomycos* or chromomycos* ) OR AB ( actinomycetoma* or maduromycos* or eumycetoma* or mycetoma* or chromoblastomycos* or chromomycos* ) | 2,677 |
| S39 | TI ( (madura or dermatitis or mossy or pedrosoi or phialophora) N4 (foot or feet or verruc* or fonsecaea) ) OR AB ( (madura or dermatitis or mossy or pedrosoi or phialophora) N4 (foot or feet or verruc* or fonsecaea) ) | 786 |
| S38 | TI ( (wuchereria or filaria* or wucheria or microfilaria* or brugia or brugoa) N4 (lymph* or bancrofti* or brugian or malay* or bancrofti or sanguinis or timori) ) OR AB ( (wuchereria or filaria* or wucheria or microfilaria* or brugia or brugoa) N4 (lymph* or bancrofti* or brugian or malay* or bancrofti or sanguinis or timori) ) | 8,177 |
| S37 | TI elephantias* OR AB elephantias* | 831 |
| S36 | (MH "Elephantiasis, Filarial") | 329 |
| S35 | TI ( lepro* or hansenias* ) OR AB ( lepro* or hansenias* ) | 11,592 |
| S34 | TI ( (hansen* or lepra* or lepro* or icrc) N4 (disease* or graecorum or infect* or morbus or bacill* or mycoblasma) ) OR AB ( (hansen* or lepra* or lepro* or icrc) N4 (disease* or graecorum or infect* or morbus or bacill* or mycoblasma) ) | 3,571 |
| S33 | (MH "Hansen's Disease") | 670 |
| S32 | TI leishmania* OR AB leishmania* | 40,068 |
| S31 | (MH "Leishmania") | 10 |
| S30 | (MH "Leishmaniasis") | 29,823 |
| S29 | TI ( nelavan or nagana ) OR AB ( nelavan or nagana ) | 207 |
| S28 | TI ( trypanosom* N4 (brucei or infect* or gambiense* or rhodesiense*) ) OR AB ( trypanosom* N4 (brucei or infect* or gambiense* or rhodesiense*) ) | 15,972 |
| S27 | TI ( (sleeping or african or negro or gambian or rhodesian or congo) N4 (sickness* or letharg* or trypanosom*) ) OR AB ( (sleeping or african or negro or gambian or rhodesian or congo) N4 (sickness* or letharg* or trypanosom*) ) | 6,012 |
| S26 | (MH "Trypanosomiasis") | 15,654 |
| S25 | TI lung N2 fluke* OR AB lung N2 fluke* | 352 |
| S24 | TI ( clonorchi* or opisthorchi* or Fasciol* or Paragonim* ) OR AB ( clonorchi* or opisthorchi* or Fasciol* or Paragonim* ) | 12,273 |
| S23 | (MH "Paragonimiasis") | 1,497 |
| S22 | (MH "Opisthorchiasis") | 1,616 |
| S21 | (MH "Fascioliasis") | 3,496 |
| S20 | (MH "Clonorchiasis") | 1,620 |
| S19 | TI ( echinococc* or hydatid* ) OR AB ( echinococc* or hydatid* ) | 16,802 |
| S18 | (MH "Echinococcosis") OR (MH "Echinococcosis, Hepatic") | 12,710 |
| S17 | TI ( dracuncul* or dracontiasis or guineaworm ) OR AB ( dracuncul* or dracontiasis or guineaworm ) | 1,490 |
| S16 | TI ( (guinea or medina or gorstius or dragon or serpent) N4 (worm* or medinens*) ) OR AB ( (guinea or medina or gorstius or dragon or serpent) N4 (worm* or medinens*) ) | 454 |
| S15 | (MH "Dracunculiasis") | 893 |
| S14 | TI ( CHIKUNGUNYA or CHIcKUNGUNYA or CHKV ) OR AB ( CHIKUNGUNYA or CHIcKUNGUNYA or CHKV ) | 7,431 |
| S13 | (MH "Chikungunya Fever") OR (MH "Chikungunya Virus") | 2,075 |
| S12 | TI ( DENV or dengue ) OR AB ( DENV or dengue ) | 29,676 |
| S11 | TI ( (breakbone* or break-bone* or dandy or denque or aden or bouquet or solar or sun) N4 (fever* or classical or virus* or disease* or infect* or flavivirus*) ) OR AB ( (breakbone* or break-bone* or dandy or denque or aden or bouquet or solar or sun) N4 (fever* or classical or virus* or disease* or infect* or flavivirus*) ) | 238 |
| S10 | (MH "Dengue") | 23,220 |
| S9 | TI ((Trypanosom or Schizotrypanum) N4 cruz* ) OR AB ( (Trypanosoma or Schizotrypanum) N4 cruz* ) | 14,476 |
| S8 | TI american N4 trypanosom* OR AB american N4 trypanosomias* | 810 |
| S7 | TI chagas* N4 disease* OR AB chagas* N4 disease* | 12,341 |
| S6 | (MH "Trypanosomiasis") | 15,654 |
| S5 | TI (Mycobacteri* N4 (ulcer* or infection* or buruli*) ) OR AB ( Mycobacteri* N4 (ulcer*or infect* or buruli*) ) | 4,169 |
| S4 | TI ( (buruli* or bairnsdale or daintree or searls) N4 (ulcer* or disease*) ) OR AB ( (buruli* or bairnsdale or daintree or searls) N4 (ulcer* or disease*) ) | 977 |
| S3 | (MH "Buruli Ulcer") | 736 |
| S2 | TI neglect* N4 disease* OR AB neglect* N4 disease* | 8,370 |
| S1 | (MH "Neglected Diseases") | 1,363 |
|  |  |  |

**SCOPUS (817)**

(

( TITLE-ABS-KEY ( neglect* W/4 disease* ) )

OR ( TITLE-ABS-KEY ( ( buruli* OR bairnsdale OR daintree OR searls ) W/4 ( ulcer* OR disease* ) ) )

OR ( TITLE-ABS-KEY ( mycobacteri* W/4 ( ulcer* OR infect* OR buruli* ) ) )

OR ( TITLE-ABS-KEY ( american W/4 trypanosom* ) )

OR ( TITLE-ABS-KEY ( chagas* W/4 disease* ) )

OR ( TITLE-ABS-KEY ( ( trypanosom* OR schizotrypanum ) W/4 cruz* ) )

OR ( TITLE-ABS-KEY ( ( ( breakbone* OR break-bone* OR dandy OR denque OR aden OR bouquet OR solar OR sun ) W/4 ( fever* OR classical OR virus* OR disease* OR infect* OR flavivirus* ) ) ) )

OR ( TITLE-ABS-KEY ( denv OR dengue* ) )

OR ( TITLE-ABS-KEY ( chikungunya OR chickungunya OR chkv ) )

OR ( TITLE-ABS-KEY ( ( guinea OR medina OR gorstius OR dragon OR serpent ) W/4 ( worm* OR medinens* ) ) )

OR ( TITLE-ABS-KEY ( dracuncul* OR dracontiasis OR guineaworm ) )

OR ( TITLE-ABS-KEY ( echinococc* OR hydatid* ) )

OR ( TITLE-ABS-KEY ( clonorchi* OR opisthorchi* OR fasciol* OR paragonim* ) )

OR ( TITLE-ABS-KEY ( lung W/2 fluke* ) )

OR ( TITLE-ABS-KEY ( ( sleeping OR african OR negro OR gambian OR rhodesian OR congo ) W/4 ( sickness* OR letharg* OR trypanosom* ) ) )

OR ( TITLE-ABS-KEY ( trypanosom* W/4 ( brucei OR infect* OR gambiense* OR rhodesiense* ) ) )

OR ( TITLE-ABS-KEY ( nelavan OR nagana ) )

OR ( TITLE-ABS-KEY ( leishmania* ) )

OR ( TITLE-ABS-KEY ( ( hansen* OR lepra* OR lepro* OR icrc ) W/4 ( disease* OR graecorum OR infect* OR morbus OR bacill* OR mycoblasma ) ) )

OR ( TITLE-ABS-KEY ( lepro* OR hansenias* ) )

OR ( TITLE-ABS-KEY ( elephantias* ) )

OR ( TITLE-ABS-KEY ( ( wuchereria OR filaria* OR wucheria OR microfilaria* OR brugia OR brugoa ) W/4 ( lymph* OR bancrofti* OR brugian OR malay* OR bancrofti OR sanguinis OR timori ) ) )

OR ( TITLE-ABS-KEY ( ( madura OR dermatitis OR mossy OR pedrosoi OR phialophora ) W/4 ( foot OR feet OR verruc* OR fonsecaea ) ) )

OR ( TITLE-ABS-KEY ( actinomycetoma* OR maduromycos* OR eumycetoma* OR mycetoma* OR chromoblastomycos* OR chromomycos* ) )

OR ( TITLE-ABS-KEY ( noma OR nomas ) )

OR ( TITLE-ABS-KEY ( ( cancrum OR stomatiti* OR mouth OR oral ) W/4 ( oris OR gangrenous OR denture OR ulcer* OR epitheli* OR mucosa OR recurrent OR granulomatosa OR virus* ) ) )

OR ( TITLE-ABS-KEY ( volvulos* OR oncho* ) )

OR ( TITLE-ABS-KEY ( river W/4 blindness ) )

OR ( TITLE-ABS-KEY ( rabies OR raby OR hydrophobia OR lyssa* OR hubert OR rabbia ) )

OR ( TITLE-ABS-KEY ( scabie* ) )

OR ( TITLE-ABS-KEY ( ( watchmaker OR sarcopt* OR seven ) W/4 ( mange OR scabi* OR itch* ) ) )

OR ( TITLE-ABS-KEY ( ( snail* OR mason* ) W/4 ( fever* OR disease* ) ) )

OR ( TITLE-ABS-KEY ( bilharzia* OR katayama OR schistoma* OR schistosom* OR bilharzios* OR schistomiasis OR haematobi* OR mansoni ) )

OR ( TITLE-ABS-KEY ( ascari* OR roundworm* OR lumbricoides OR trichuri* OR whipworm* OR trichocephal* OR hookworm* OR ancylostoma* OR necator* ) )

OR ( TITLE-ABS-KEY ( ( soil OR transmitted ) W/3 helminth* ) )

OR ( TITLE-ABS-KEY ( snakebite* ) )

OR ( TITLE-ABS-KEY ( snake* W/4 ( bite* OR envenom* ) ) )

OR ( TITLE-ABS-KEY ( taenia* OR cysticercos* OR cysticercus OR coenur* OR cysticerciasis ) )

OR ( TITLE-ABS-KEY ( ( tapeworm* OR solium* ) W/4 ( infect* OR pork* ) ) )

OR ( TITLE-ABS-KEY ( trachoma* ) )

OR ( TITLE-ABS-KEY ( ( granular OR egyptian ) W/4 ( conjunctivitis OR ophthalmia ) ) )

OR ( TITLE-ABS-KEY ( frambesia* OR yaw* OR framboesia* OR parangi OR pian OR pertenue OR treponema OR pallid* ) )

)

AND

( TITLE-ABS-KEY ( acceptabilit* ) )
